# Inequalities in Maternal Health Care Utilization in Sub-Saharan African Countries: A Multiyear and Multi-Country Analysis

**DOI:** 10.1371/journal.pone.0120922

**Published:** 2015-04-08

**Authors:** Nazmul Alam, Mohammad Hajizadeh, Alexandre Dumont, Pierre Fournier

**Affiliations:** 1 Research Centre of the University of Montreal Hospital (CR-CHUM), Montreal, Quebec, Canada; 2 School of Public Health, University of Montreal, Montreal, Canada; 3 School of Health Administration, Dalhousie University, Halifax, Canada; 4 Research Institute for Development, Université Paris Descartes, Paris, France; Institute for Health & the Environment, UNITED STATES

## Abstract

To assess social inequalities in the use of antenatal care (ANC), facility based delivery (FBD), and modern contraception (MC) in two contrasting groups of countries in sub-Saharan Africa divided based on their progress towards maternal mortality reduction. Six countries were included in this study. Three countries (Ethiopia, Madagascar, and Uganda) had <350 MMR in 2010 with >4.5% average annual reduction rate while another three (Cameroon, Zambia, and Zimbabwe) had >550 MMR in 2010 with only <1.5% average annual reduction rate. All of these countries had at least three rounds of Demographic and Health Surveys (DHS) before 2012. We measured rate ratios and differences, as well as relative and absolute concentration indices in order to examine within-country geographical and wealth-based inequalities in the utilization of ANC, FBD, and MC. In the countries which have made sufficient progress (i.e. Ethiopia, Madagascar, and Uganda), ANC use increased by 8.7, 9.3 and 5.7 percent, respectively, while the utilization of FBD increased by 4.7, 0.7 and 20.2 percent, respectively, over the last decade. By contrast, utilization of these services either plateaued or decreased in countries which did not make progress towards reducing maternal mortality, with the exception of Cameroon. Utilization of MC increased in all six countries but remained very low, with a high of 40.5% in Zimbabwe and low of 16.1% in Cameroon as of 2011. In general, relative measures of inequalities were found to have declined overtime in countries making progress towards reducing maternal mortality. In countries with insufficient progress towards maternal mortality reduction, these indicators remained stagnant or increased. Absolute measures for geographical and wealth-based inequalities remained high invariably in all six countries. The increasing trend in the utilization of maternal care services was found to concur with a steady decline in maternal mortality. Relative inequality declined overtime in countries which made progress towards reducing maternal mortality.

## Introduction

Sub-Saharan Africa (SSA) has the highest maternal mortality ratio in the world and account for more than half of maternal death worldwide [1]. Despite major progress towards reducing maternal mortality over the last decade, it is unlikely that many countries in the world, including the SSA region, will be able to achieve their Millennium Development Goal of 75% decrease in maternal mortality by 2015 [2,3]. Several countries in SSA experience civil war and social conflict that jeopardize overall development and social stability. Others are relatively conflict free, but are still lagging behind their desired progress in terms of health indicators.

Lack of access to health care services for pregnancy and delivery are among the main reasons for high maternal and neonatal mortality rates worldwide [4,5]. Maternal care services remain important indicators for monitoring the progress of maternal outcomes, including maternal mortality. Antenatal care, delivery at health facilities with skilled professionals, and postnatal care reinforce the timely management and treatment of complications to reduce maternal deaths. In spite of the importance of facility based delivery in preventing maternal death, a larger proportion of women give birth outside of health facilities without any skilled attendance [6,7]. Antenatal care is one of the pillars of the Safe Motherhood Initiative, and helps provide interventions that are necessary for healthy pregnancy outcomes [8,9]. Receiving antenatal care at least four times, as recommended by the WHO, increases the likelihood of receiving effective health promotion and preventive maternal health interventions during antenatal visits [2,10,11]. Family planning is another important indicator of the Safe Motherhood Initiative to reduce maternal death in developing countries [10]. Basic and comprehensive emergency obstetric services are recommended as lifesaving interventions for pregnant women [12,13]. It is important to assess whether the utilization of these key services is equitable across countries, and by geographic and socioeconomic strata within them.

The United Nations report on ‘Trends in Maternal Mortality 2005–2010’ categorizes the countries based on their progress towards MDG 5 targets from 1900 to 2010 [2]. One important feature of this report is that it demonstrates progress by providing the average annual percentage change in maternal mortality ratio (MMR) between 1990 and 2010, and categorizes the countries as being ‘on track’, ‘making progress’, ‘insufficient progress’ or ‘no progress’ made over time. We identified six countries from the SSA region based on the WHO report on maternal mortality. Three of these countries *viz*. Ethiopia, Madagascar, and Uganda had <350 MMR in 2010, made sufficient progress towards achieving their MDG 5 targets, maintained an average annual reduction rate of >4.5, and had available Demographic and Health Surveys (DHS) data for 2010 or 2011. The other three countries *viz*. Cameroon, Zambia and Zimbabwe had >550 MMR in 2010, made insufficient or no progress towards achieving their MDG 5 targets, maintained an average annual reduction rate of <1.5, and had DHS data available for 2010 or 2011.

Two research questions were addressed in this study: how three key maternal health indicators (antenatal care, delivery at health facilities and use modern contraception) were utilized in the better performing countries and poor performing countries in SSA against the backdrop of MMR status over time?, and whether there were geographic and wealth-based inequalities in the utilization of these services in these countries? We hypothesize that utilization of maternal care services is higher and more equitable by urban-rural and wealth based gradients in better performing countries compared to poor performing countries. We undertook comparative analyses of utilization of antenatal care, delivery at health facilities and modern contraception in six selected countries in general and separately by urban-rural and wealth quintiles to assess inequalities in the utilization of these services.

## Materials and Methods

Data collected from the Demographic and Health Surveys in six SSA countries were used in this study. DHS surveys are cross-sectional, comparable and nationally representative surveys of household samples that collect information on various health and demographic issues including sections on maternal and child health, fertility and family planning from selected low-and-middle-income countries (LMICs) [14]. We used three rounds of the latest DHS surveys available between 1994 and 2011 in six selected countries and labelled them as Group A: Ethiopia (ET), Madagascar (MD), and Uganda (UG); and Group B: Cameroon (CM), Zambia (ZM), and Zimbabwe (ZW). This grouping was conducted based on the data presented in the report ‘Trends in Maternal Mortality 2005–2010’ published jointly by the WHO and other UN organizations, which categorizes countries based on their progress towards meeting MDG 5 targets from 1990 to 2010. Table 1 presents the survey year and sample size for all DHSs included in the study.

**Table 1:** Countries, survey year and sample size for all DHS included in the study.

Countries (Group A)	Survey Year	Sample size/HH	Sample size/Women Aged 15–49	Countries (Groups B)	Survey Year	Sample size/HH	Sample size/Women Aged 15–49
**Ethiopia (ET)**				**Cameroon (CM)**			
	2000	14072	15367		1998	4697	5501
	2005	13721	14070		2004	10462	10656
	2011	16702	16515		2011	14214	15426
**Madagascar (MD)**				**Zambia (ZM)**			
	1997	7171	7060		1996	7286	8021
	2003/04	8420	7949		2001/02	7126	7658
	2008/09	17857	17375		2007	7164	7146
**Uganda (UG)**				**Zimbabwe (ZW)**			
	2000/01	7885	7246		1999	6369	5907
	2006	8870	8531		2005/06	9285	8907
	2011	9033	8674		2010/11	9756	9171

HH = household.

### Measures

The primary outcome variables of interest in the study were antenatal care (ANC), facility based delivery (FBD) and use of modern contraceptives (MC). DHS used information from mothers (aged 15–49) who had live births in the five years preceding the survey to measure ANC and FBD. As per the WHO's recommendation, we calculated ANC as having a minimum of four prenatal care visits during their pregnancy. FBD is defined as giving birth at a health facility such as a public or private hospital, community health center or private clinic. MC was examined for women (aged 15–49 years) who reported active use of a modern method of contraception, such as the oral pill, intrauterine device, condom, female or male sterilization, implant or injectable. Two key exploratory variables used in this study to analyse inequalities in the utilization of maternal and reproductive care services (i.e., ANC, FBD and MC). These variables are place of residence (rural-urban) and household wealth index. Using a method suggested by Filmer and Pritchett, [15] the DHS constructs the wealth index based on available information about households asset ownership, and housing and environmental conditions [16]. The MMR remains as the centre of interest of this study and the six countries were selected based on their performance in achieving their target to reduce maternal mortality. MMR refers to the number of women who die during pregnancy or within 42 days of delivery per 100,000 live births. MMR is a regression modeled estimate developed by the WHO, UNICEF, UNFPA and the World Bank. MMR is estimated using information on fertility, birth attendants, and HIV prevalence [2].

### Statistical Analysis

We calculated utilization of three maternal and reproductive health measures (ANC, FBD and MC) for each of the six selected countries as whole, for rural and urban areas and for two extreme wealth quintiles for three DHS surveys to present as proportions. Then, to measure absolute and relative differences between urban and rural areas in the terms of utilization of maternal and reproductive health care, we used rate ratios (RR_ur_ = R_urban_÷R_rural_) and rate differences (RD_ur_ = R_urban_-R_rural_), respectively. We also measured the rate ratios of the highest vs. lowest wealth quintile (RR_hl_ = R_highest quintile_÷ R_lowest quintile_.), as well as the difference between the ratios of the highest and lowest wealth quintile (RD_hl_ = R_highest quintile_- R_lowest quintile_) to calculate socioeconomic inequalities in maternal and reproductive health care. Additionally, we used relative and absolute measures of the concentration index to quantify socioeconomic inequalities in ANC, FBD and MC. The relative concentration index (RC) was measured with reference to the relative concentration curve. The relative concentration curve requires the plotting of the cumulative population of individuals, ranked in ascending order of socioeconomic status (such as household wealth) on its x-axis, against the cumulative percentage of the health variable of interest on its y-axis. The concentration curve enables us to make statements such as “10% of ANC was used by the poorest 20% of the population”. The RC is defined as twice the area between the relative concentration curve and the diagonal line representing “the line of perfect equality”. If the relative concentration curve lies below (above) the line of perfect equality, the RC takes a negative (positive) value and means that the health variable is concentrated among wealthier individuals [17]. The value of RC ranges from -1 to 1, with a score of zero indicating “no disparity”. We can also generalize the concentration curve in such a way that it becomes sensitive to changes in the population mean of the health variable (*μ*), and therefore reflects absolute differences in health outcomes across socioeconomic groups. The generalized concentration curve is the relative concentration curve multiplied by the health variable *μ*. The absolute concentration index (AC) is twice the area between the line of perfect equality and the generalized concentration curve. AC is calculated as *μ*×*RC* and ranges from -*μ* to *μ*, with zero indicating “perfect equality” [18]. The RC and AC indices were calculated using the “convenient regression” approach suggested by Kakwani et al. (1997). Moreover, as the outcome variables of interest in our study (i.e. ANC, FBD and MC) are binary, we applied Wagstaff’s correction [19] to the measurement of RC and AC (i.e. multiplying the RC by 1/1-*μ*). We used a method suggested by Altman and Bland [20] to examine the significance of differences in the values of RR_ur_, RD_ur_, RR_hl,_ RD_hl_, RC and AC between the first and last surveys at the p-value = 0.05 level with 95% confidence intervals.

## Results

### Maternal Mortality Ratio in selected countries

**Fig 1:**
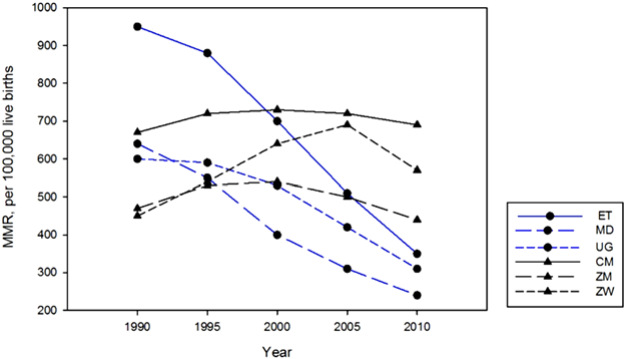
Maternal mortality ratio (per 100,000 live births) in selected SSA countries: 1990–2010.

There were two different trends in the MMR across the six selected SSA countries over the period between 1990 and 2010 (Fig. 1). The MMR declined in Ethiopia, Madagascar and Uganda over the last two decades whereas the MMR increased or plateaued during the same period in Zimbabwe, Zambia and Cameroon. Ethiopia witnessed a more dramatic decline in maternal mortality over this time period, with an MMR of 950/per 100,000 live births in 1990 and 350/per 100,000 live births in 2010. By contrast, the MMR in Zimbabwe increased from 470/per 100,000 deaths in 1990 to 570/per 100,000 deaths in 2010.

### Trends in the utilization of ANC, FBD and MC services

**Fig 2:**
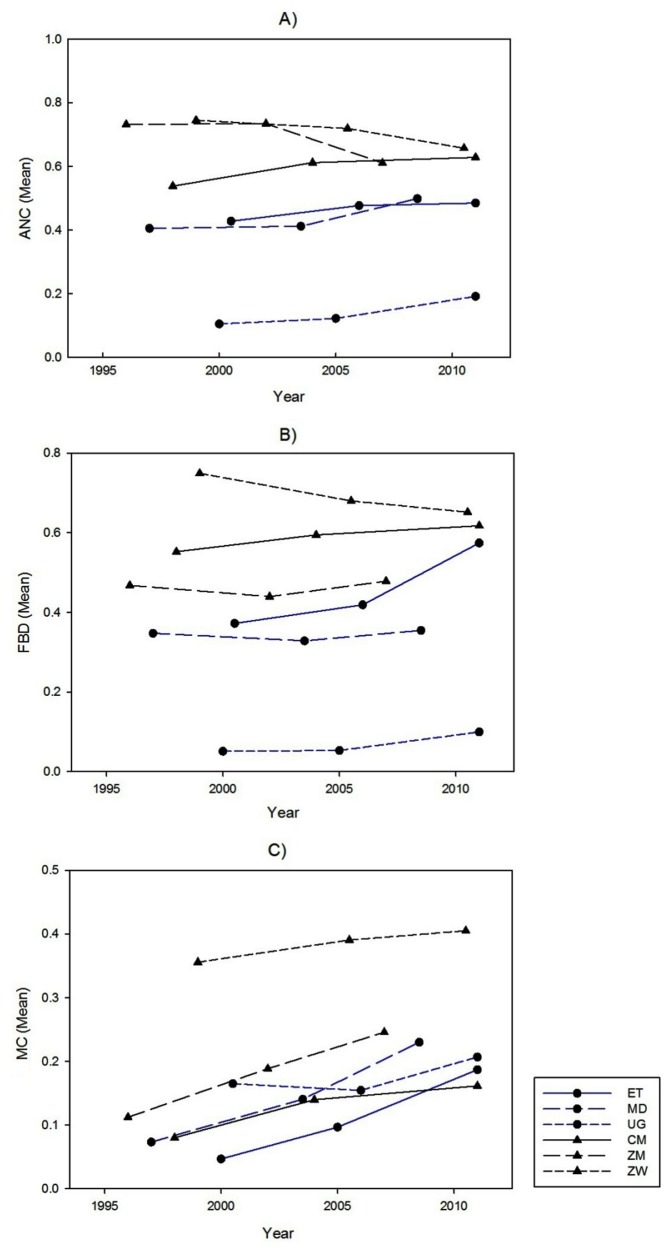
Proportion of antenatal care (ANC), facility based delivery (FBD) and modern contraceptive use (MC) in selected countries.

The utilization of ANC increased in the three countries with sufficient progress towards their MDG 5 target (Fig. 2). The difference in the proportions of ANC use between the first and third surveys suggested 8.7, 9.3 and 5.7 percent increases in Ethiopia, Madagascar and Uganda, respectively (Table 2). With the exception of Uganda, where the proportion of ANC use increased from 39.2 to 46.6 percent over time in rural areas only, the utilization of ANC increased in both rural and urban regions in countries with satisfactory progress towards their MDG 5 goals. The utilization of FBD increased over time in both rural and urban areas in Ethiopia as well as in Uganda. The utilization of FBD did not change over time in Madagascar. Results also indicated that modern contraceptive use increased by 14, 15.7 and 4.2 percent over time in Ethiopia, Madagascar and Uganda, respectively. Among countries with insufficient progress towards their MDG 5 targets, only Cameroon showed increased utilization of maternal and reproductive health care. Although the uptake of modern contraceptive methods increased by 13.3 and 4.9 percent in Zambia and Zimbabwe over time, the utilization of ANC declined in these countries. Furthermore, in Zimbabwe, the utilization of FBD declined from 75 percent in 1999 to 65 percent in 2010/11.

**Table 2:** Rates and ratios showing changes in urban-rural disparities in antenatal care (ANC), facility based delivery (FBD) and modern contraceptive (MC) use in countries with sufficient (A) and insufficient (B) progress for maternal mortality.

	ANC				FBD				MC			
Country	Survey 1	Survey 2	Survey 3	Survey 3-Survey 1	Survey 1	Survey 2	Survey 3	Survey 3-Survey 1	Survey 1	Survey 2	Survey 3	Survey 3-Survey 1
**A. Ethiopia**	**2000**	**2005**	**2011**		**2000**	**2005**	**2011**		**2000**	**2005**	**2011**	
** Total**	0.105	0.122	0.191	0.087 (0.071 to 0.102)	0.051	0.053	0.1	0.049 (0.039 to 0.058)	0.047	0.097	0.187	0.14 (0.13 to 0.15)
** Urban**	0.445	0.549	0.464	0.019 (-0.046 to 0.084)	0.317	0.425	0.499	0.182 (0.128 to 0.236)	0.146	0.19	0.256	0.11 (0.082 to 0.138)
** Rural**	0.062	0.082	0.144	0.082 (0.068 to 0.096)	0.02	0.024	0.041	0.021 (0.014 to 0.028)	0.025	0.077	0.165	0.14 (0.13 to 0.15)
** RR_ur_**	7.21	6.729	3.218	-3.992 (-5.237 to -2.747)	15.893	18.043	12.28	-3.613 (-7.518 to 0.292)	5.886	2.472	1.55	-4.336 (-5.482 to 3.19)
** RD_ur_**	0.384	0.468	0.319	-0.065 (-0.132 to 0.002)	0.297	0.402	0.458	0.161 (0.106 to 0.216)	0.121	0.113	0.091	-0.03 (-0.06 to 0.001)
**A. Madagascar**	**1997**	**2003/04**	**2008/09**		**1997**	**2003/04**	**2008/09**		**1997**	**2003/04**	**2008/09**	
** Total**	0.405	0.412	0.499	0.093 (0.072 to 0.115)	0.347	0.328	0.355	0.007 (-0.012 to 0.027)	0.073	0.14	0.23	0.157 (0.147 to 0.167)
** Urban**	0.518	0.564	0.719	0.201 (0.154 to 0.248)	0.414	0.428	0.604	0.19 (0.145 to 0.235)	0.123	0.195	0.257	0.134 (0.114 to 0.154)
** Rural**	0.377	0.376	0.468	0.091 (0.068 to 0.114)	0.331	0.306	0.324	-0.007 (-0.028 to 0.014)	0.054	0.122	0.224	0.17 (0.159 to 0.181)
** RR_ur_**	1.372	1.501	1.537	0.165 (0.021 to 0.309)	1.251	1.399	1.863	0.612 (0.447 to 0.777)	2.295	1.598	1.148	-1.147 (-1.549 to -0.745)
** RD_ur_**	0.14	0.188	0.251	0.111 (0.058 to 0.164)	0.083	0.122	0.28	0.197 (0.147 to 0.247)	0.07	0.073	0.033	-0.037 (-0.06 to -0.014)
**A. Uganda**	**2000/01**	**2006**	**2011**		**2000/01**	**2006**	**2011**		**2000/01**	**2006**	**2011**	
** Total**	0.428	0.477	0.485	0.057 (0.033 to 0.08)	0.372	0.419	0.574	0.202 (0.184 to 0.22)	0.165	0.154	0.207	0.042 (0.028 to 0.056)
** Urban**	0.686	0.612	0.584	-0.102 (-0.152 to -0.052)	0.796	0.793	0.895	0.099 (0.07 to 0.128)	0.329	0.282	0.285	-0.044 (-0.076 to-0.012)
** Rural**	0.392	0.457	0.466	0.074 (0.048 to 0.1)	0.321	0.371	0.521	0.2 (0.181 to 0.219)	0.132	0.129	0.187	0.055 (0.04 to 0.07)
** RR_ur_**	1.751	1.339	1.255	-0.496 (-0.647 to -0.345)	2.475	2.136	1.718	-0.757 (-0.893 to -0.621)	2.493	2.189	1.52	-0.973 (-1.273 to -0.673)
** RD_ur_**	0.294	0.155	0.119	-0.175 (-0.232 to -0.118)	0.474	0.422	0.374	-0.1 (-0.134 to -0.066)	0.197	0.153	0.098	-0.099 (-0.135 to -0.063)
**B. Cameroon**	**1998**	**2004**	**2011**		**1998**	**2004**	**2011**		**1998**	**2004**	**2011**	
** Total**	0.538	0.612	0.629	0.091 (0.066 to 0.115)	0.552	0.595	0.618	0.065 (0.042 to 0.089)	0.08	0.14	0.161	0.081 (0.072 to 0.091)
** Urban**	0.685	0.756	0.776	0.091 (0.055 to 0.127)	0.826	0.82	0.853	0.027 (-0.001 to 0.055)	0.137	0.201	0.218	0.081 (0.064 to 0.098)
** Rural**	0.484	0.488	0.506	0.022 (-0.009 to 0.053)	0.45	0.419	0.445	-0.005 (-0.034 to 0.024)	0.049	0.066	0.095	0.046 (0.035 to 0.057)
** RR_ur_**	1.416	1.548	1.533	0.117 (-0.002 to 0.236)	1.838	1.957	1.917	0.079 (-0.057 to 0.215)	2.794	3.057	2.294	-0.5 (-1.066 to 0.066)
** RD_ur_**	0.201	0.268	0.27	0.069 (0.021 to 0.117)	0.377	0.401	0.408	0.031 (-0.01 to 0.072)	0.088	0.135	0.123	0.035 (0.015 to 0.055)
**B. Zambia**	**1996**	**2001/02**	**2007**		**1996**	**2001/02**	**2007**		**1996**	**2001/02**	**2007**	
** Total**	0.732	0.734	0.611	-0.121 (-0.14 to -0.101)	0.468	0.44	0.478	0.011 (-0.007 to 0.029)	0.112	0.188	0.246	0.133 (0.12 to 0.147)
** Urban**	0.819	0.818	0.598	-0.221 (-0.255 to-0.187)	0.771	0.793	0.839	0.068 (0.043 to 0.093)	0.17	0.273	0.271	0.101 (0.078 to 0.124)
** Rural**	0.675	0.691	0.617	-0.058 (-0.082 to -0.034)	0.266	0.282	0.329	0.063 (0.044 to 0.082)	0.065	0.132	0.227	0.162 (0.147 to 0.177)
** RR_ur_**	1.214	1.184	0.969	-0.245 (-0.312 to -0.178)	2.896	2.812	2.55	-0.346 (-0.545 to -0.147)	2.617	2.065	1.195	-1.422 (-1.794 to -1.05)
** RD_ur_**	0.145	0.127	-0.019	-0.164 (-0.205 to -0.123)	0.505	0.511	0.51	0.005 (-0.026 to 0.036)	0.105	0.141	0.044	-0.061 (-0.088 to -0.034)
**B. Zimbabwe**	**1999**	**2005/06**	**2010/11**		**1999**	**2005/06**	**2010/11**		**1999**	**2005/06**	**2010/11**	
** Total**	0.745	0.719	0.657	-0.088 (-0.113 to -0.063)	0.749	0.68	0.652	-0.098 (-0.118 to -0.077)	0.356	0.391	0.405	0.049 (0.032 to 0.067)
** Urban**	0.752	0.774	0.671	-0.081 (-0.13 to -0.032)	0.912	0.931	0.851	-0.061 (-0.091 to -0.031)	0.419	0.411	0.391	-0.028 (-0.059 to 0.003)
** Rural**	0.742	0.694	0.651	-0.091 (-0.119 to -0.063)	0.669	0.578	0.567	-0.102 (-0.128 to -0.076)	0.316	0.377	0.414	0.098 (0.078 to 0.118)
** RR_ur_**	1.015	1.115	1.03	0.015 (-0.066 to 0.096)	1.362	1.61	1.501	0.139 (0.062 to 0.216)	1.326	1.088	0.944	-0.382 (-0.497 to -0.267)
** RD_ur_**	0.011	0.08	0.02	0.009 (-0.048 to 0.066)	0.242	0.353	0.284	0.042 (0.002 to 0.082)	0.103	0.033	-0.023	-0.126 (-0.163 to -0.089)

RR_ur_ = R_urban_÷R_rural_, RD_ur_ = R_urban_-R_rural_. Data in parentheses are 95% confidence intervals.

### Inequalities in the utilization of ANC, FBD and MC services

The calculated RR_ur_ and RD_ur_ suggested marked relative and absolute urban-rural disparities between the utilization of ANC, FBD and MC in all countries (see Table 2). As for the urban-rural inequality, Ethiopia made considerable reduction in relative disparities in ANC use between the first and third surveys, a 55 percent reduction in the value of the RR_ur_. While relative and absolute urban-rural inequalities in the utilization of ANC and FBD increased in Madagascar over time, the RR_ur_ and RD_ur_ suggest a decline in geographical differences in ANC and FBD in Uganda. Among countries with insufficient reduction in maternal care, Zambia witnessed a decrease in urban-rural differences in terms of utilization of all measures of maternal and reproductive health services (see the significant declines in the values of the estimated RR_ur_ and RD_ur_ for ANC, FBD and MC between the first and third surveys for Zambia in Table 2). In contrast, geographical differences in the absolute utilization of ANC in Cameroon increased by 0.069 (CI = 0.021 to 0.117) points and absolute and relative utilization of FBD in Zimbabwe widened over time by 0.139 (CI = 0.062 to 0.216) and 0.042 (CI = 0.002 to 0.082) points, respectively.

Based on the results of the rich-to-poor ratios (RR_hl_) and absolute differences between the richest and poorest quintiles (RD_hl_), it is evident that wealthier mothers utilized more ANC, FBD and MC in all countries (Table 3). The calculated RR_hl_ and RD_hl_ demonstrated huge variations in disparities of maternal and reproductive health care between mothers in the wealthiest and poorest quintiles across all the countries. According to the RD_hl_, the absolute inequalities in the utilization of ANC and FBD widened in all countries over time except for in Zambia, where the absolute gaps in ANC and FBD use decreased between the first and last surveys by 0.201 (CI = -0.264 to -0.138) and 0.075 (CI = -0.115 to -0.035) points, respectively. The rich-poor ratios in the utilization of FBD and/or ANC also increased in Madagascar, Cameroon and Zimbabwe. In contrast to Madagascar, Zambia and Zimbabwe, the absolute and relative inequalities in MC use between women in the wealthiest and poorest quintiles increased over time Uganda by 0.124 (0.083 to 0.165) points and 1.28 (0.71 to 1.85) points, accordingly.

**Table 3:** Rates and ratios showing changes in wealth-based disparities in antenatal care (ANC), facility based delivery (FBD) and modern contraceptive (MC) use in countries with sufficient (A) and insufficient (B) progress for maternal mortality.

	ANC				FBD				MC			
Country	Survey 1	Survey 2	Survey 3	Survey 3-Survey 1	Survey 1	Survey 2	Survey 3	Survey 3-Survey 1	Survey 1	Survey 2	Survey 3	Survey 3-Survey 1
**A. Ethiopia**	**2000**	**2005**	**2011**		**2000**	**2005**	**2011**		**2000**	**2005**	**2011**	
** Lowest WQ**	0.041	0.04	0.083	0.042 (0.02 to 0.064)	0.007	0.006	0.02	0.013 (0.004 to 0.022)	0.019	0.03	0.102	0.083 (0.066 to 0.1)
** Highest WQ**	0.353	0.396	0.467	0.114 (0.06 to 0.168)	0.23	0.247	0.45	0.22 (0.176 to 0.264)	0.132	0.178	0.261	0.129 (0.104 to 0.154)
** RR_hl_**	8.681	9.839	5.633	-3.048 (-6.227 to 0.131)	30.822	43.494	22.129	-8.693 (-30.964 to 13.578)	6.965	5.901	2.561	-4.404 (-7.155 to -1.653)
** RD_hl_**	0.312	0.355	0.384	0.072 (0.013 to 0.131)	0.222	0.241	0.43	0.208 (0.163 to 0.253)	0.113	0.148	0.159	0.046 (0.016 to 0.076)
** RC**	0.533	0.523	0.45	-0.083 (-0.135 to -0.031)	0.71	0.738	0.701	-0.009 (-0.057 to 0.039)	0.494	0.354	0.201	-0.293 (-0.34 to -0.246)
** AC**	0.056	0.064	0.086	0.03 (0.023 to 0.037)	0.036	0.039	0.07	0.034 (0.031 to 0.037)	0.023	0.034	0.038	0.015 (0.011 to 0.019)
**A. Madagascar**	**1997**	**2003/04**	**2008/09**		**1997**	**2003/04**	**2008/09**		**1997**	**2003/04**	**2008/09**	
** Lowest WQ**	0.329	0.294	0.352	0.023 (-0.016 to 0.062)	0.258	0.195	0.178	-0.08 (-0.112 to -0.048)	0.02	0.065	0.154	0.134 (0.117 to 0.151)
** Highest WQ**	0.664	0.732	0.769	0.105 (0.052 to 0.158)	0.451	0.546	0.665	0.214 (0.162 to 0.266)	0.158	0.209	0.265	0.107 (0.083 to 0.131)
** RR_hl_**	2.018	2.488	2.184	0.166 (-0.12 to 0.452)	1.749	2.798	3.742	1.993 (1.561 to 2.425)	7.707	3.23	1.724	-5.983 (-9.187 to -2.779)
** RD_hl_**	0.335	0.438	0.417	0.082 (0.016 to 0.148)	0.193	0.351	0.488	0.295 (0.234 to 0.356)	0.137	0.145	0.111	-0.026 (-0.055 to 0.003)
** RC**	0.225	0.327	0.307	0.082 (0.037 to 0.127)	0.212	0.324	0.415	0.203 (0.16 to 0.246)	0.429	0.27	0.13	-0.299 (-0.347 to -0.251)
** AC**	0.091	0.135	0.153	0.062 (0.042 to 0.082)	0.074	0.106	0.147	0.073 (0.058 to 0.088)	0.031	0.038	0.03	-0.001 (-0.006 to 0.004)
**A. Uganda**	**2000/01**	**2006**	**2011**		**2000/01**	**2006**	**2011**		**2000/01**	**2006**	**2011**	
** Lowest WQ**	0.421	0.448	0.431	0.01 (-0.043 to 0.063)	0.353	0.28	0.423	0.07 (0.029 to 0.111)	0.14	0.063	0.107	-0.033 (-0.061 to -0.005)
** Highest WQ**	0.448	0.662	0.61	0.162 (0.114 to 0.21)	0.391	0.764	0.877	0.486 (0.456 to 0.516)	0.194	0.281	0.285	0.091 (0.061 to 0.121)
** RR_hl_**	1.064	1.48	1.414	0.35 (0.16 to 0.54)	1.106	2.726	2.076	0.97 (0.789 to 1.151)	1.385	4.453	2.665	1.28 (0.71 to 1.85)
** RD_hl_**	0.027	0.215	0.179	0.152 (0.08 to 0.224)	0.037	0.484	0.455	0.418 (0.367 to 0.469)	0.054	0.218	0.178	0.124 (0.083 to 0.165)
** RC**	-0.002	0.144	0.143	0.145 (0.097 to 0.193)	-0.029	0.364	0.343	0.372 (0.335 to 0.409)	0.014	0.345	0.221	0.207 (0.161 to 0.253)
** AC**	-0.001	0.069	0.069	0.07 (0.048 to 0.092)	-0.011	0.152	0.197	0.208 (0.19 to 0.226)	0.002	0.053	0.046	0.044 (0.036 to 0.052)
**B. Cameroon**	**1998**	**2004**	**2011**		**1998**	**2004**	**2011**		**1998**	**2004**	**2011**	
** Lowest WQ**	0.34	0.398	0.338	-0.002 (-0.051 to 0.047)	0.259	0.28	0.172	-0.087 (-0.128 to -0.046)	0.015	0.03	0.027	0.012 (0.001 to 0.023)
** Highest WQ**	0.756	0.873	0.875	0.119 (0.075 to 0.163)	0.859	0.918	0.958	0.099 (0.065 to 0.133)	0.156	0.253	0.263	0.107 (0.083 to 0.131)
** RR_hl_**	2.226	2.196	2.592	0.366 (0.003 to 0.729)	3.309	3.277	5.558	2.249 (1.542 to 2.956)	10.169	8.512	9.759	-0.41 (-6.411 to 5.591)
** RD_hl_**	0.417	0.475	0.538	0.121 (0.055 to 0.187)	0.599	0.638	0.786	0.187 (0.134 to 0.24)	0.14	0.223	0.236	0.096 (0.07 to 0.122)
** RC**	0.357	0.428	0.463	0.106 (0.052 to 0.16)	0.535	0.588	0.695	0.16 (0.109 to 0.211)	0.418	0.416	0.362	-0.056 (-0.106 to -0.006)
** AC**	0.192	0.262	0.291	0.099 (0.069 to 0.129)	0.296	0.35	0.429	0.133 (0.104 to 0.162)	0.033	0.058	0.059	0.026 (0.021 to 0.031)
**B. Zambia**	**1996**	**2001/02**	**2007**		**1996**	**2001/02**	**2007**		**1996**	**2001/02**	**2007**	
** Lowest WQ**	0.641	0.63	0.597	-0.044 (-0.084 to -0.004)	0.196	0.203	0.285	0.089 (0.059 to 0.119)	0.044	0.089	0.251	0.207 (0.179 to 0.235)
** Highest WQ**	0.878	0.866	0.633	-0.245 (-0.294 to -0.196)	0.91	0.915	0.924	0.014 (-0.013 to 0.041)	0.209	0.317	0.29	0.081 (0.049 to 0.113)
** RR_hl_**	1.369	1.374	1.06	-0.309 (-0.42 to -0.198)	4.64	4.506	3.242	-1.398 (-1.898 to -0.898)	4.771	3.558	1.154	-3.617 (-4.704 to -2.53)
** RD_hl_**	0.237	0.236	0.036	-0.201 (-0.264 to -0.138)	0.714	0.712	0.639	-0.075 (-0.115 to -0.035)	0.165	0.228	0.039	-0.126 (-0.169 to -0.083)
** RC**	0.239	0.255	0.033†	-0.206 (-0.253 to -0.159)	0.614	0.584	0.51	-0.104 (-0.14 to -0.068)	0.361	0.323	0.074	-0.287 (-0.336 to -0.238)
** AC**	0.175	0.187	0.02†	-0.155 (-0.186 to -0.124)	0.287	0.257	0.244	-0.043 (-0.06 to -0.026)	0.041	0.061	0.018	-0.023 (-0.032 to -0.014)
**B. Zimbabwe**	**1999**	**2005/06**	**2010/11**		**1999**	**2005/06**	**2010/11**		**1999**	**2005/06**	**2010/11**	
** Lowest WQ**	0.741	0.651	0.612	-0.129 (-0.178 to -0.08)	0.599	0.457	0.463	-0.136 (-0.181 to -0.091)	0.305	0.34	0.412	0.107 (0.07 to 0.144)
** Highest WQ**	0.775	0.825	0.748	-0.027 (-0.085 to 0.031)	0.952	0.947	0.899	-0.053 (-0.082 to -0.024)	0.431	0.381	0.371	-0.06 (-0.098 to -0.022)
** RR_hl_**	1.046	1.267	1.223	0.177 (0.058 to 0.296)	1.59	2.074	1.942	0.352 (0.19 to 0.514)	1.412	1.121	0.901	-0.511 (-0.69 to -0.332)
** RD_hl_**	0.034	0.174	0.136	0.102 (0.026 to 0.178)	0.353	0.49	0.436	0.083 (0.029 to 0.137)	0.126	0.041	-0.041	-0.167 (-0.22 to -0.114)
** RC**	0.009†	0.152	0.106	0.097 (0.034 to 0.16)	0.365	0.491	0.402	0.037 (-0.016 to 0.09)	0.135	0.029	-0.026	-0.161 (-0.2 to -0.122)
** AC**	0.007†	0.109	0.07	0.063 (0.018 to 0.108)	0.273	0.334	0.262	-0.011 (-0.049 to 0.027)	0.048	0.011	-0.01	-0.058 (-0.073 to -0.043)

WQ = wealth quintile. RR_hl_ = R_highest quintile_÷ R_lowest quintile_. RD_hl_ = R_highest quintile_- R_lowest quintile_. RC = relative concentration index. AC = absolute concentration index. Data in parentheses are 95% confidence intervals. All the RC/AC indices are statistically significantly different from zero at the 5 percent level except for the indices denoted.

Moreover, the results demonstrated significant rich-poor disparities in the consumption of maternal health services in all six countries (see positive values of the RC and AC indices in Table 3). According to the RC and AC indices, while over time absolute and relative wealth-based inequalities in ANC and FBD use decreased in Zambia, such disparities increased in Madagascar, Uganda and Cameroon. The estimated values of RC and AC indices in Zimbabwe also indicated that wealth-based inequities in the utilization of ANC increased by 0.097 (0.034 to 0.16) points and 0.063 (0.018 to 0.108) points, accordingly. Wealth-based inequalities in the utilization of reproductive health care decreased in Madagascar, and in the three countries with insufficient progress towards their MDG 5 targets. In Ethiopia, while absolute wealth-based inequalities in the utilization of maternal and reproductive health care increased between the first and last surveys, relative wealth-based inequalities in ANC and MC use decreased over time.

## Discussion

The results of this study showed that coverage of ANC and FBD has steadily increased over the last decade in countries that have made progress towards reducing maternal mortality (Ethiopia, Madagascar, and Uganda). By contrast, with the exception of Cameroon, utilization of those services in countries which have not made sufficient progress towards reducing maternal mortality either plateaued or decreased. In general, relative measures of inequality, such as rate ratios and relative concentration index, demonstrated a decline in inequalities of the utilization of maternal care in countries which have made satisfactory progress towards reducing maternal mortality. At the same time, these indicators suggested sustained or even increased trends in inequalities in countries with insufficient progress towards reducing maternal mortality. Absolute measures for geographical and wealth-based inequalities, however, remained invariably high in all six countries.

An increase in the proportion of women who received ANC and FBD over time concurred with a steady decline in maternal mortality. This finding was remarkable since these two measures are considered to be crucial in reducing maternal mortality [4,21]. Similar findings have been reported in some other countries, and the declining trend in maternal mortality ratios suggests that the uptake of maternity care services could have a strong impact on MMR reduction [22,23]. Uptake of ANC and FBD services may contribute to identifying and addressing life-threatening maternal conditions which lead to maternal mortality [8]. Increase in uptake of ANC, FBD and MC services in both rural and urban areas but more pragmatically in rural areas in countries made sufficient progress towards reducing maternal mortality is a reflection in reduction of relative inequality.

Direct causes of maternal mortality are haemorrhages, hypertensive disorders, sepsis, obstructed labour, and complications from unsafe abortions. However, use of contraception remains as one of the primary interventions for the prevention of maternal mortality [10,11,21,24]. Use of modern contraceptives was found to be very low in all six countries included in this study. According to the 2011 survey, the highest was reported at 40.5% in Zimbabwe and the lowest at 16.1% in Cameroon. The highest changes were chiefly observed in Ethiopia and Madagascar; two countries which have made sufficient progress towards achieve the MDG 5 targets: their utilization of MC increased by 14% and 16%, respectively. Nevertheless, in order to make further improvement in meeting maternal mortality targets, all of the studied countries need to make further boosts to family planning campaigns. A very low rate of contraceptive use has been observed in Cameroon, only 16.1 percent as of 2011 DHS survey. Apparently no progress has been made in contraceptive use rates during last decade even though the country has made some progress in the utilization of ANC and FBD rates compared to Zambia and Zimbabwe. Role of HIV epidemic and poor nutritional status among women may have contributed in high maternal mortality in SSA countries including Zambia and Zimbabwe [25–27].

Many barriers to the access of maternal health and reproductive health services have been identified in LMICs, including the African context. These factors included affordability of services, availability of services, distance to the services, lack of transportation, sociocultural factors and the lack of knowledge [24,28–31]. ‘The three delays model’ provided a conceptual framework of the factors influencing access to appropriate care in obstetric emergencies [32].

Our findings demonstrated that pregnant women in urban areas used more ANC, FBD and MC services in all countries. Ethiopia has the highest level of relative inequalities in the utilization of these services, but the country has nevertheless achieved considerable reduction in urban-rural disparities, thus making progress towards reducing maternal mortality. However, it needs to be noted here that Ethiopia had the lowest initial utilization rates of ANC, FBD and MC among the six countries, providing sufficient room for improvement comparatively. Wealth-based inequalities as measured on an absolute scale suggested that wealthier mothers utilized more ANC, FBD and MC in all countries. These findings support the existing literature, which also shows evidence for substantial inequalities in the use of reproductive and maternal health in terms of wealth and rural-urban settings in various African and Asian countries [7,8,33–38]. Explaining wealth-based inequality in accessing maternal and reproductive care services is complex, because many factors, such as cost versus affordability, distance and availability of services, sociocultural factors, and demand for services must be considered [37,39,40]. Lack of affordability can be seen as a factor that explains rich-poor inequalities for ANC, FBD and MC services [39,40]. Demand for services influenced by cultural factors negatively affect poorer women to seek care maternal and reproductive health services, this may be because they are less educated, they prefer to delivery at home, do not want to see a male doctor [41,42] Urban-rural differentials in maternal care utilization could be explained by the concentration of health infrastructure and better quality services in urban areas [43,44].

To handle inequality in the utilization of maternal health services, strategies that encompass both demand and supply side interventions, especially to reach those living in remote areas or with limited resources, are a necessity. Such interventions should go beyond any targeted intervention for maternal health to accommodate a broader development agenda, including human capital development. For example, investment in female education may have contributed in reduction of maternal mortality in countries like Ethiopia and Bangladesh [5,45]. Ethiopia has put an additional three million children in school than in 2000 through rural school construction programmes. In Bangladesh, girls' enrolment in primary school is 92.3% [45]. Free or subsidized maternal care, as in the cases of Ghana, Burkina Faso [46], voucher schemes in Uganda, Cambodia, and Bangladesh [47–49], transport interventions in Malawi and Burundi, [50,51] are remarkable. Lessons learned from implementation of these highly applauded interventions must be contextualised to increase the use of maternal care services, and to address the disparities in the accessing maternal care services in SSA countries in order to achieve their targets in reduction of maternal mortality.

There are a few limitations to this study. First, the three DHS surveys for the six countries may not have been strictly comparable because they were conducted across a range of different years, from 1996 to 2011. However, the total duration that the three surveys refer to remain more or less similar, around 10 years. Second, there was a recall period of utilization of care ranging from four to six years between the surveys, but the wealth index was constructed on data from the survey year. Nevertheless, current measures of wealth index can be an acceptable proxy of immediately past years since household wealth is a relatively stable attribute that does not change in the short term. Third, the inclusion of other important indicators to maternal care, in particular emergency obstetric care (EmOC), would have been highly relevant in order to provide a degree of comparability with the MMR status of the countries. However, information on EmOC is not readily available, except for caesarean section delivery. We did not analyze inequalities in the utilization of caesarean sections because the available samples were insufficient for conducting statistical inference [12]. We have selected the six countries based on certain criteria, one of them being the availability of DHS survey data for multiple rounds; hence, caution is needed to interpret and generalize the study's findings.

## Conclusion

This study has pointed out the importance of key maternal care services and related socioeconomic and geographic inequality against the backdrop of maternal mortality status in six SSA countries. The findings revealed persistent inequalities in the use of three key maternal health services, favouring wealthy and urban women. Relative inequality by wealth and rural-urban residence decreased over time in countries making progress towards reducing maternal mortality, but it was not the case in countries made insufficient progress. Although our findings highlighted the importance of disparities in maternal care services on MMR in the selected countries, it should be noted that other factors such as governance, socioeconomic, infrastructural and environmental conditions play an important role in reduction of disparities and hence MMR in each country [37,42,43]. Thus, the findings presented in this paper should be the subject of further research in order to establish a causal relationship between the utilization of care and maternal mortality. As we strive for universal health coverage, future health policies and interventions must be strengthened to increase the use of maternal care services, and be more able to address the disparities in the utilization of maternal care, especially for those who are poor and live in rural areas in SSA countries. It is through this kind of action that these countries can hope to achieve the MDG targets and beyond.
